# Stem cells in middle ear cholesteatoma contribute to its pathogenesis

**DOI:** 10.1038/s41598-018-24616-4

**Published:** 2018-04-18

**Authors:** Julia Nagel, Saskia Wöllner, Matthias Schürmann, Viktoria Brotzmann, Janine Müller, Johannes FW Greiner, Peter Goon, Barbara Kaltschmidt, Christian Kaltschmidt, Holger Sudhoff

**Affiliations:** 1 Department of Otolaryngology, Head and Neck Surgery, Klinikum Bielefeld, 33604 Bielefeld, Germany; 2 Department of Cell Biology, University of Bielefeld, 33619 Bielefeld, Germany; 3 Department of Dermatology, Addenbrooke’s Hospital, Hills Road, Cambridge, CB22 7PP UK; 4 AG Molecular Neurobiology, University of Bielefeld, 33619 Bielefeld, Germany

## Abstract

Cholesteatoma is a potentially life-threatening middle ear lesion due to the formation of an inflamed ectopic mass of keratinizing squamous epithelium. Surgical removal remains the only treatment option, emphasizing the need to gain a better understanding of this severe disease. We show for the first time that stem cells residing in cholesteatoma tissue contribute to disease progression. Cells expressing the “stemness” markers Nestin and S100B were detected in middle ear cholesteatoma and auditory canal skin. Isolated Nestin + /S100B + -cells showed the capability for self-renewal, neurosphere formation and differentiation into mesodermal and ectodermal cell types. Compared to auditory canal skin stem cells middle ear cholesteatoma-derived stem cells displayed an enhanced susceptibility to inflammatory stimuli, and this suggested a possible contribution to the inflammatory environment in cholesteatoma tissue. Cholesteatoma derived stem cells were able to differentiate into keratinocyte-like cells using factors mimicking the microenvironment of cholesteatoma. Our findings demonstrate a new perspective on the pathogenesis of cholesteatoma and may lead to new treatment strategies for this severe middle ear lesion.

## Introduction

Cholesteatoma is an expanding lesion of the middle ear, consisting of stratified keratinizing squamous epithelium. Typical clinical symptoms comprise hearing loss, ear discharge and ear pain^1^. Its locally invasive growth pattern may result in the destruction of pivotal structures within the temporal bone. Even though osteoneogenesis is one of the symptoms of cholesteatoma, squamous epithelium may be rendered destructive in an environment of chronic infection, thereby also triggering osteolytic effects. In northern Europe there are approximately 9.2 new cases in 100,000 people per year^1^ whereas the risk of a cholesteatoma is higher for male patients^2^. 16.9% of all patients show bilateral cholesteatomas^3^. To date, medical management strategies are limited (reviewed in^4^) and surgical removal is the only possible treatment option for cholesteatomas^5^. Antibiotics and antimycotics can only treat cholesteatomatous otitis media and superinfections before surgery, thereby reducing skin re-growth and post-surgical complications^6^.

Cholesteatomas can be classified into congenital and acquired cholesteatoma^7^. While congenital cholesteatoma represent only 2–4% of all cases^8^ in children at the age of 4–6 years, acquired cholesteatomas are found in children and adults. Different theories exist regarding the origin and pathogenesis of cholesteatoma (reviewed in^9^). Cholesteatoma development comprises several biological and molecular processes involving cell migration, proliferation, extracellular matrix deposition, and tissue remodelling. Notably, hyperproliferative mucosal tissue like nasal polyps as well as endometriosis and atherosclerotic lesions were shown to contain stem cell populations^10,11^. In atherosclerotic lesions, the formation particularly involves migration of stem cells from bone marrow and the vascular wall into the lesion^12^. To investigate their potential role in the middle ear cholesteatoma, we analyzed cholesteatoma tissue and auditory canal skin for the presence of stem cells. Our findings demonstrate, for the first time, the presence of a stem cell population in cholesteatoma tissue and auditory canal skin. Furthermore the stem cells derived from the cholesteatoma showed a higher expression of the Toll-like receptor 4 (TLR4) and a higher susceptibility to inflammatory stimulus *in-vitro* in comparison to stem cells derived from healthy auditory canal skin. Factors present in the middle ear cholesteatoma microenvironment were also able to differentiate the cholesteatoma-derived stem cells into epidermal cell types.

## Results

### Cells expressing the stem cell marker Nestin are present in middle ear cholesteatoma tissue and auditory canal skin

**Figure 1:**
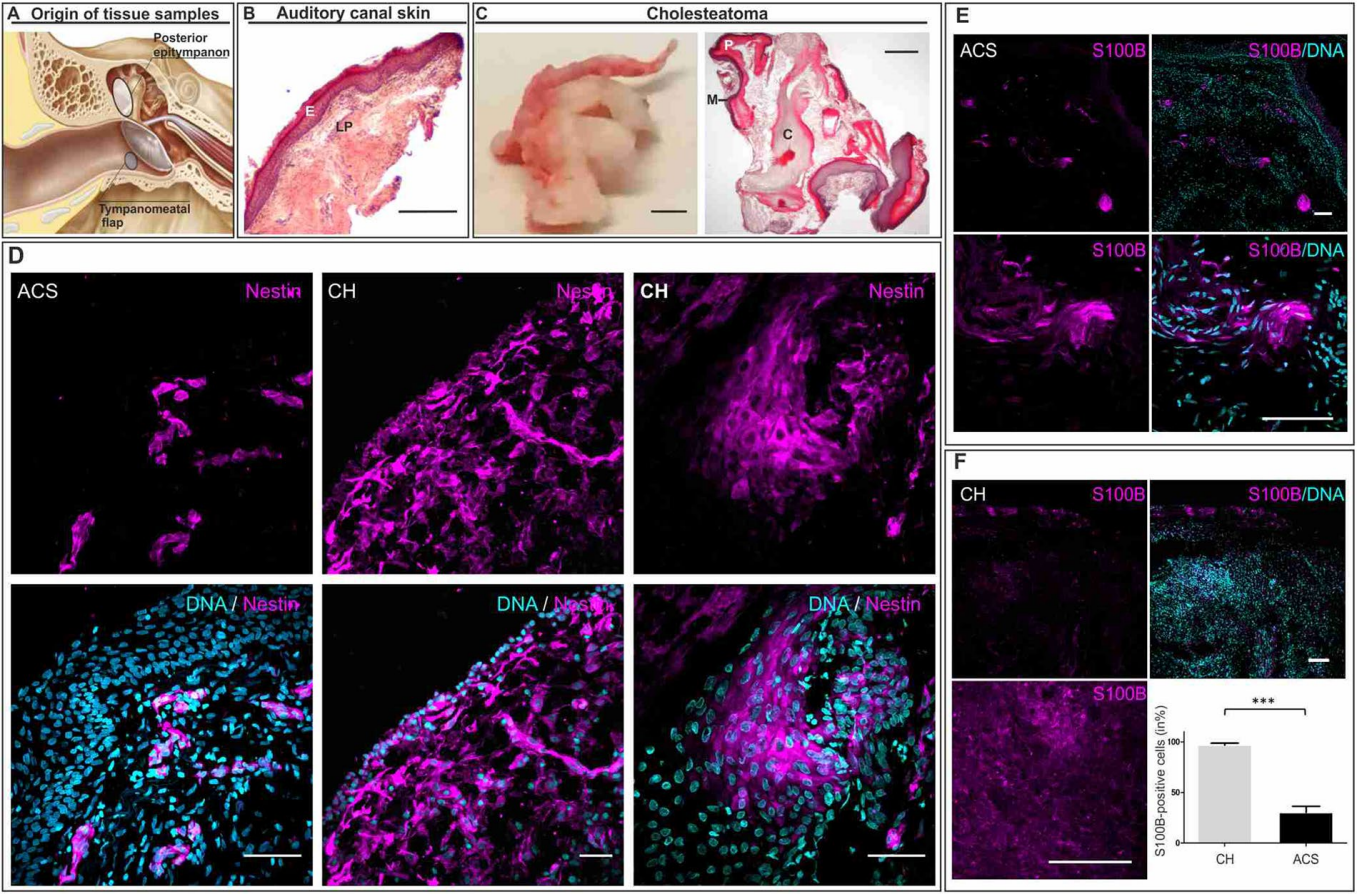
*Cholesteatoma tissue and auditory canal skin reveal the presence of Nestin- and S100B- positive cells*. (**A**) Localization of cholesteatoma (CH) and auditory canal skin (ACS) harvested during middle ear surgery. (**B**) H&E staining of ACS showing the epithelial layer (E) and lamina propria (LP). Scale bar: 500 µm. (**C**) Cholesteatoma tissue (CH) revealed characteristic structures of matrix (M), perimatrix (P), and cystic contents (C) in a H&E-staining. Scale bar: 1 cm. (**D**) Immunohistochemical staining of sectioned ACS showed nestin-positive cells located in the lamina propria, whereas sectioned cholesteatoma tissue revealed nestin-positive cells in homogenous or cluster-like distributions. Scale bar: 50 µm. (**E**) Immunohistochemical analysis of ACS indicated the expression of S100B positive cells within the lamina propria. Scale bar: 100 µm.(**F**) The number of S100B-positive cells in the CH is significantly higher compared to the ACS (Five arbitrary scanned fields were analysed, ***p < 0.001 was considered significant, unpaired t-test, two tailed, confidence interval: 95%). Scale bar: 100 µm.

The cholesteatoma tissue was routinely extracted from the posterior epitympanon. The auditory canal skin samples were dissected from the tympano-meatal flap, resulting from middle ear surgery (Fig. 1A). We investigated morphology using Haematoxylin and Eosin (H&E) staining, and we demonstrated the characteristic epithelial layer and lamina propria of the auditory canal skin (Fig. 1B) as well as the characteristic structures of matrix (M), perimatrix (P), and cystic contents (C) in cholesteatoma tissue (Fig. 1C). Using immunohistochemical analysis, cells expressing the stem cell marker Nestin were detected in the auditory canal skin, located within the lamina propria and within the matrix and perimatrix of middle ear cholesteatoma tissue (Fig. 1D). We further detected cells positive for the neural crest marker S100B in the lamina propria of the auditory canal skin. A significantly higher amount of S100B-positive cells was observed in cholesteatoma tissue in comparison to healthy auditory canal skin (Fig. 1E–F). In addition, co-localization of S100B and Nestin was observable in cells residing within cholesteatoma tissue and auditory canal skin (Supplementary Figure S1). The appropriate negative controls are shown in the Supplementary Figure S2.

### Middle Ear Cholesteatoma derived Stem Cells (ME-CSCs) can be successfully isolated and cultivated under serum-free conditions

**Figure 2:**
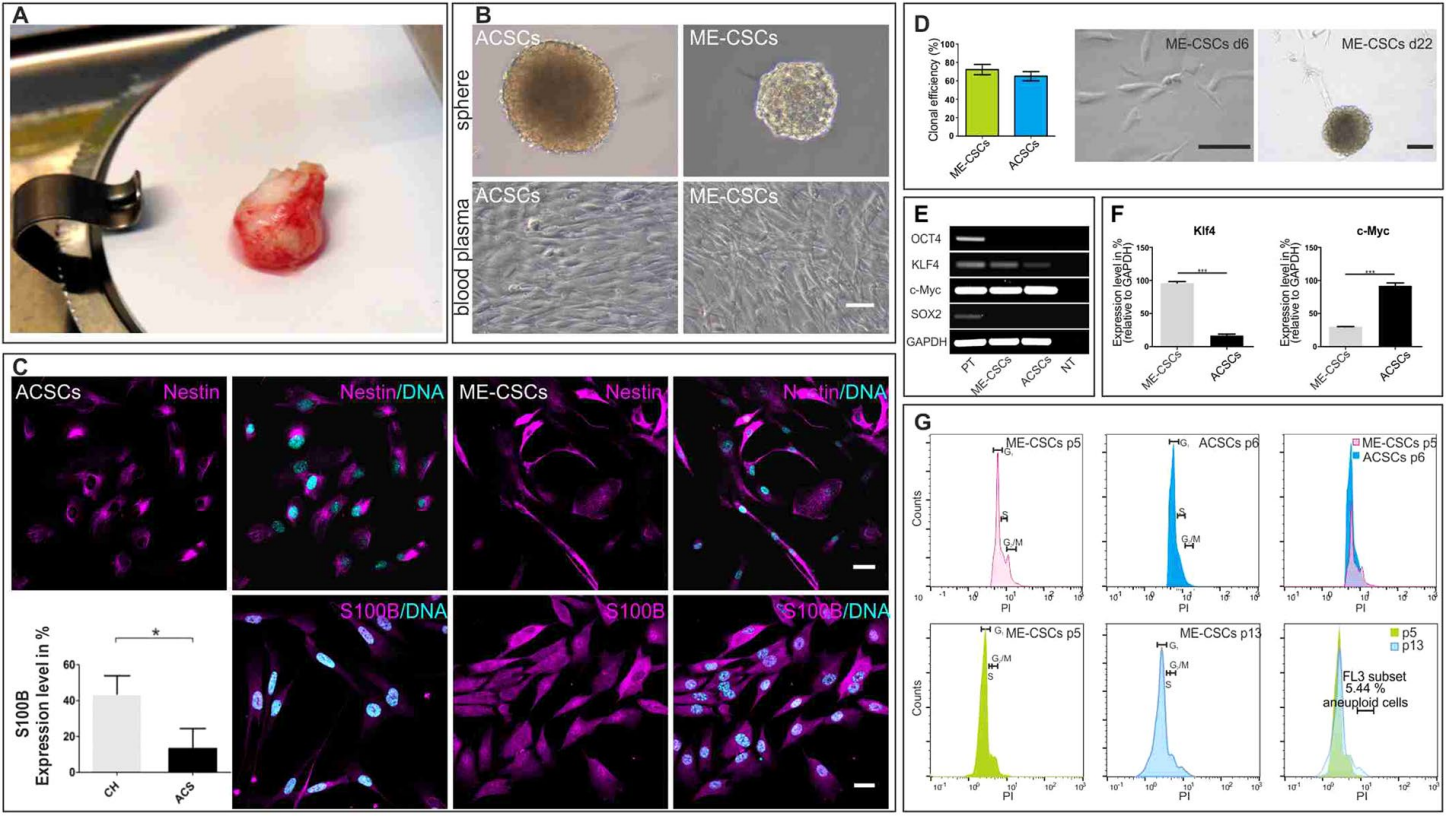
Nestin-expressing cells from cholesteatoma tissue (ME-CSCs) and auditory canal skin (ACSCs) can be cultivated *in vitro* while showing stem cell characteristics and a stable DNA content. (**A**) Surgically removed cholesteatoma. (**B**) Light microscopic images of cells isolated from auditory canal skin (ACSCs) and middle ear cholesteatoma-derived stem cells (ME-CSCs**)**, which can be cultivated as spheres (upper panels) and in a human blood plasma-based 3D-fibrin matrix thereby exhibiting a long-shaped morphology (lower panels). Scale bar: 100 µm. (**C**) Cultivated ACSCs and ME-CSCs showed the expression of Nestin at protein-level and biological triplicates demonstrated a significantly higher expression of S100B in cultivated ME-CSCs at mRNA-level (qPCR Analysis). Scale bar: 20 µm (technical triplicates *p < 0.05 was considered significant, unpaired t-test, two tailed, confidence interval: 95%). (**D**) Light microscopic images of ME-CSCs, which gave rise to clonal colonies in a limited dilution assay after 6 days and gave rise to secondary spheres after 22 days of clonal growth. Scale bar: 200 µm. Statistical analyses on technical triplicates showed no significant difference in clonal efficiency between ME-CSCs and ACSCs. (**E**) Cultivated ACSCs and ME-CSCs showed the expression of the stem cell markers Oct4, Klf4, c-Myc and Sox2 on mRNA-level (RT-PCR analysis; raw data shown in Supplementary Figure S3). (NT: Non-template control. PT: Positive control, iPSCs.). (**F**) Real time qPCR analysis of the stem cell marker Klf4 and c-Myc in cultivated ME-CSCs and ACSCs. (technical triplicates ***p < 0.001 was considered significant, unpaired t-test, two tailed, confidence interval: 95%). (**G**) PI staining with subsequent flow cytometric analysis showed typical DNA-content for diploid cells without signs of polyploidy in ME-CSCs at passage 5 and in ACSCs at passage 6. Only 5.44% potentially aneuploid cells in ME-CSCs at passage 13 were detected.

After mechanical and enzymatic dissociation, cells were successfully isolated from middle ear cholesteatoma tissue (Fig. 2A) and auditory canal skin. Successfully isolated cells showed the ability to form spheres under serum-free culture conditions in the presence of epidermal growth factor (EGF) and basic fibroblast growth factor (bFGF; also known as FGF2 or FGF-β) (Fig. 2B, upper panels). Such putative middle ear cholesteatoma derived stem cells (ME-CSCs) and auditory canal skin stem cells (ACSCs) were expandable within a human blood plasma-based fibrin matrix, thereby revealing long spindle-shaped cell bodies (Fig. 2B, lower panels).

### Cultivated ME-CSCs express neural crest and stemness markers and show the ability for self-renewal

Upon investigation of the expression profile of putative ME-CSCs and ACSCs, we observed expression of Nestin and S100B at a protein level by immunocytochemistry (Fig. 2C). In comparison to ACSCs, ME-CSCs showed a significantly increased expression of S100B at mRNA-level (Fig. 2C). As a hallmark of their stem cell character, ME-CSCs and ACSCs possessed the capacity for self-renewal resulting in the formation of secondary spheres after 22 days of clonal growth. We observed no significant differences in clonal efficiency between ME-CSCs and ACSCs (Fig. 2D). To further investigate the stem cell characteristics of ME-CSCs and ACSCs, the expression profiles were analyzed by reverse transcription PCR. Cultivated ME-CSCs and ACSCs showed expression of the “stemness” markers Klf4 and c-Myc, while Oct4 and Sox2 were only expressed by induced pluripotent stem cells serving as positive controls (Fig. 2E). Quantitative real-time PCR showed a significantly increased expression of Klf4 in ME-CSCs compared to ACSCs. Conversely, ME-CSCs revealed a reduced expression level of c-Myc in comparison to ACSCs (Fig. 2F).

### Cultivated ME-CSCs and ACSCs remain genetically stable

Cholesteatoma formation is described as not being associated with changes in ploidy^13^, hence genetic stability of ME-CSCs and ACSCs was analyzed. DNA content measurement via Propidium iodide (PI) staining with subsequent flow cytometric analysis depicted a typical DNA-content for diploid cells without signs of polyploidy in ME-CSCs at passage 5 and ACSCs at passage 6. Only 5.44% potentially aneuploid cells were detected in long-term cultivated ME-CSCs at passage 13 (Fig. 2G).

### Cultivated ME-CSCs and ACSCs differentiate into ectodermal and mesodermal lineages

**Figure 3:**
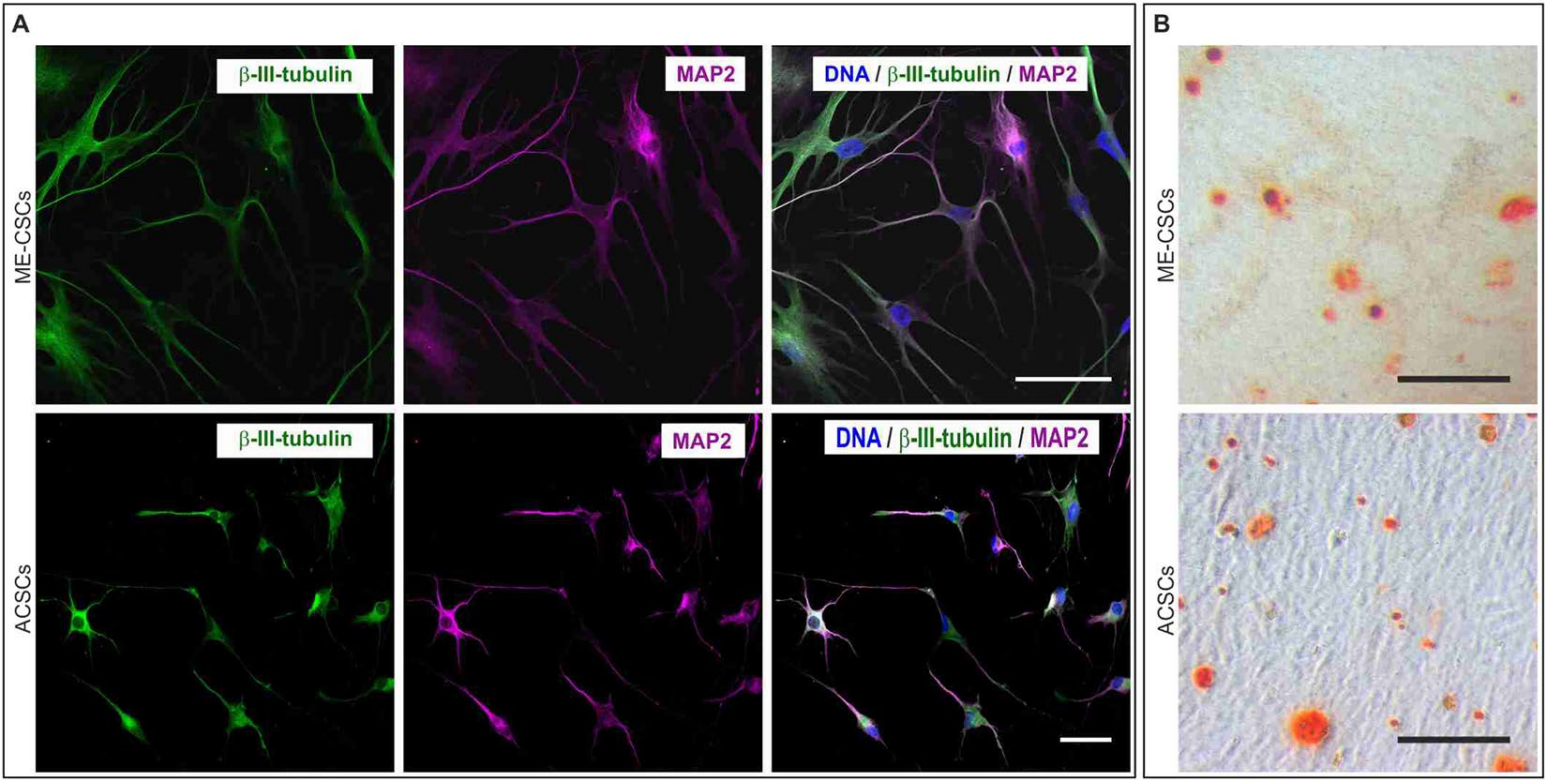
The isolated stem cells are multipotent. (**A**) ACSCs and ME-CSCs were cultivated for 28 days with neuronal induction medium and immunocytochemicaly stained and analyzed. The cells exhibited a neuronal morphology and expressed the neuronal markers anti-β-III-tubulin and MAP2. Scale bar: 50 µm. (**B**) Additionally the stem cells were treated with osteogenic induction medium for 21 days. An Alizarin red S staining visualized the deposition of calcium deposits by differentiated mature osteoblasts. Scale bar: 300 µm.

To investigate the potential of ME-CSCs and ACSCs differentiating into the ectodermal lineage, ME-CSCs were cultivated in neuronal differentiation medium for 4 weeks. After directed differentiation, ME-CSCs and ACSCs showed a characteristic neuronal morphology accompanied by expression of the neuronal markers β-III-tubulin and MAP2 at protein level (Fig. 3A). ME-CSCs and ACSCs were also exposed to osteogenic cues in a directed osteogenic differentiation assay (Fig. 3B). Alizarin red S-stained calcium deposits could be detected in ME-CSCs and ACSC-cultures after 21 days, demonstrating their successful differentiation into osteogenic cell types.

### ME-CSCs possess a higher potential to differentiate into the keratinocyte-like cells compared to ACSCs in a cholesteatoma microenvironment *in vitro*

**Figure 4:**
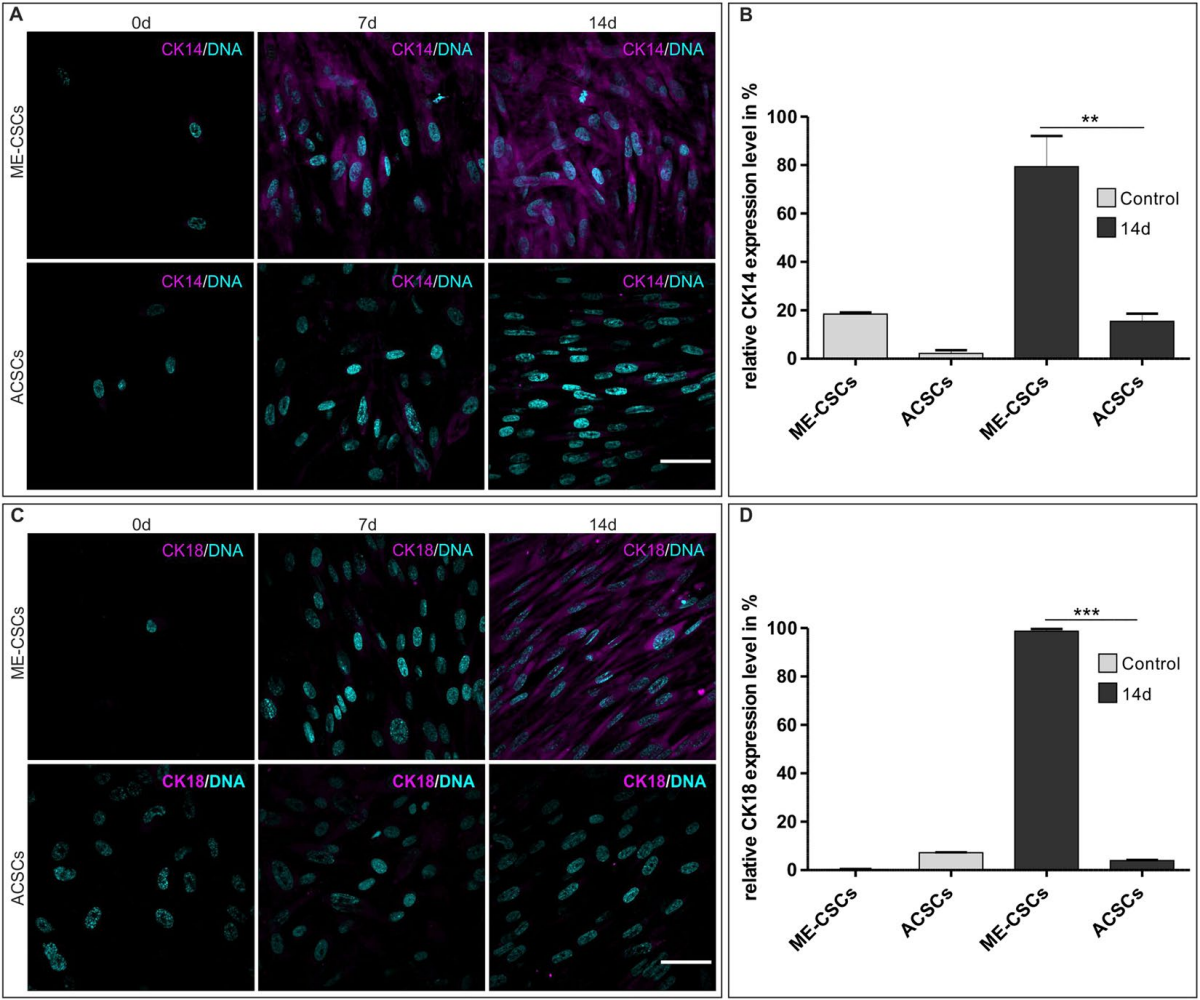
*Cultivated ME-CSCs are able to differentiate into keratinocyte-like cells* by factors mimicking the microenvironment of the cholesteatoma. (**A**,**C**) Compared to control, induced differentiation into the epithelial lineage resulted in a high expression of cytokeratin 14 and 18 in ME-CSCs after 14 days on protein level. Scale bar: 50 µm. (**B**,**D**) Real time qPCR analysis showed significantly higher expression of cytokeratin 14 and 18 in ME-CSCs, compared to ACSCs, indicating an efficient differentiation of ME-CSCs into the epithelial lineage after 14 days (technical triplicates ***p < 0.001 and **p < 0.01 was considered significant, One-way ANOVA, Bonferroni’s Multiple Comparison Test, confidence interval: 95%).

To investigate the role of ME-CSCs in cholesteatoma development and progression, we established an *in vitro* microenvironment for cholesteatoma by application of defined factors. ME-CSCs were exposed to culture media containing EGF, hepatocyte growth factor (HGF) and keratinocyte growth factor (KGF) for 14 days. Immunocytochemistry revealed strong expression of Cytokeratin 14 and 18 (CK-14 and CK-18) in ME-CSCs at day 14 of cultivation indicating efficient differentiation into the epithelial lineage. Notably, no expression of CK-14 and CK-18 was detectable at protein level in ACSCs exposed to EGF, HGF and KGF for 14 days (Fig. 4A,C). Real time PCR analyses effectively confirmed this by detection of only low mRNA-levels of CK-14 and CK-18 after 14 days of cultivation. In comparison to ACSCs, ME-CSCs showed significantly higher expression levels of CK-14 and CK-18, demonstrating that ME-CSCs possess the enhanced ability to give rise to keratinocyte-like cells (Fig. 4B,D).

### Increased expression of the Toll-like receptor 4 (TLR4) in cholesteatoma tissue is conserved in ME-CSCs with an enhanced susceptibility to inflammatory stimulus

**Figure 5:**
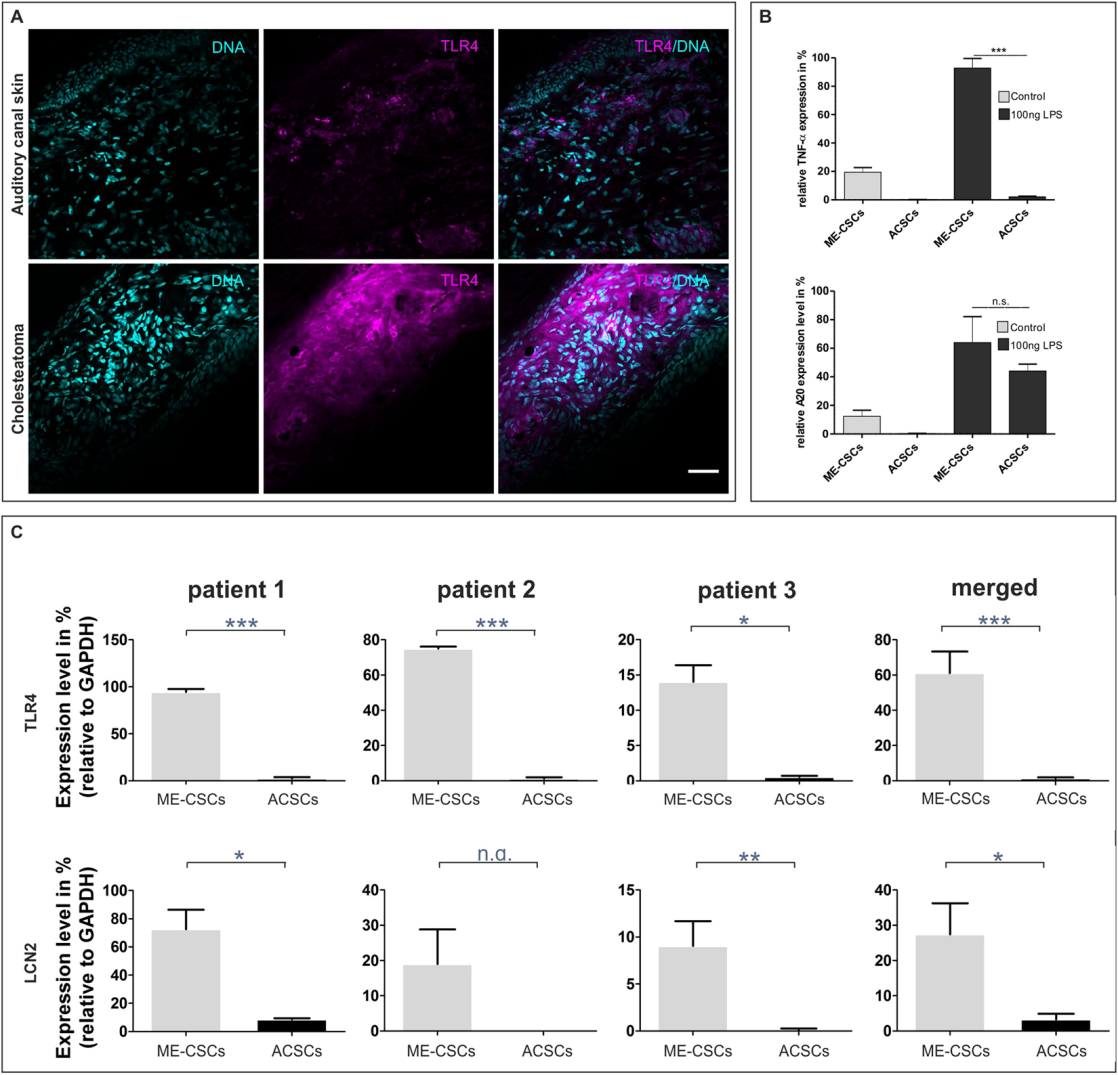
Overexpression of the Toll-like receptor 4 (TLR4) in cholesteatoma tissue is conserved in the ME-CSCs and accompanied by expression of LCN2, TNF-α and A20. (**A**) Immunohistochemical staining of the cholesteatoma tissue and ACS showed increased expression of TLR4 on protein-level in the cholesteatoma tissue in comparison to the ACS. Scale bar: 50 µm. (**B**) Real time qPCR depicted significantly increased expression levels of the pro-inflammatory genes TNF-α and A20 in ME-CSCs and an increased response after the treatment with 100 ng/ml LPS for 6 h in comparison to the ACSCs (technical triplicates ***p < 0.001 was considered significant, One-way ANOVA, Bonferroni´s Multiple Comparison Test, confidence interval: 95%). (**C**) On the mRNA-level real time PCR analysis of biological triplicates revealed significantly increased expression of the TLR4 and the innate immune protein Lipocalin2 (LCN2) in the ME-CSCs compared to the ACSCs. (***p < 0.001, **p < 0.01 and *p < 0.05 was considered significant, unpaired t-test, two tailed, confidence interval: 95%).

Since TLR4 is a crucial mediator of inflammatory signalling, we analysed cholesteatoma tissue for TLR4-expression. In accordance with previous findings^14^, immunohistochemistry of cholesteatoma tissue and auditory canal skin showed increased expression of TLR4 in cholesteatoma tissue (Fig. 5A). To investigate the conservation of this observation in ME-CSCs, we assessed expression levels of TLR4 and the innate immune protein Lipocalin2 (LCN2) using real-time PCR. Significantly increased expression of TLR4 and LCN2 transcripts could be observed in ME-CSCs compared to the ACSCs (Fig. 5C). Moreover, real-time PCR analysis showed a strongly increased expression of the pro-inflammatory mediators TNF-α and A20 in ME-CSCs in comparison to ACSCs. Simulating a bacterial infection characteristic for cholesteatoma, we observed a highly significant increase in expression of TNF-α in ME-CSCs in response to treatment with the TLR4-ligand LPS in comparison to ACSCs and untreated ME-CSCs (Fig. 5B).

## Discussion

In this study, we show for the first time the presence of stem cell populations in cholesteatoma tissue and auditory canal skin. Middle ear cholesteatoma derived stem cells (ME-CSCs) and stem cells isolated from auditory canal skin (ACSCs) were successfully isolated and cultivated *in vitro* and demonstrated their capacity for neurosphere formation, and clonal growth. Furthermore, ME-CSCs and ACSCs expressed neural crest-specific “stemness” markers, showed multipotent differentiation potentials and kept their genetic stability during cultivation.

Our findings show evenly distributed Nestin-positive cells within the lamina propria of auditory canal skin. Interestingly, Nestin-positive stem cells were likewise shown to be evenly distributed within the lamina propria of respiratory mucosa of the human nose^15^. Stem cells have also been isolated from other hyperproliferative mucosal tissue. For instance, cells isolated from nasal polyps showed mesenchymal stem cell-like characteristics *in vitro*^10^. To further investigate possible stem cell properties of these Nestin-positive cells found in auditory canal skin and cholesteatoma tissue, we established a serum-free *in vitro* cultivation method of cholesteatomaderived cells using a human plasma-based 3D fibrin matrix according to the protocols of Greiner *et al*.^16^. Successful *in vitro* culture of cholesteatoma-derived cells has already been described for fibroblasts and keratinocytes isolated from cholesteatoma tissue^17–19^. In contrast to our isolation method, cholesteatoma-derived fibroblasts are obtained using medium containing 10% fetal calf serum^19^. Addition of specific growth factors such as keratinocyte growth factor (KGF) is crucial for the successful cultivation of cholesteatoma-derived keratinocytes^18^. Furthermore, we have utilised the growth factors EGF and FGF, which have been broadly described to be essential for serum-free cultivation of adult stem cells^15,20,21^. ME-CSCs cultivated under stem cell conditions demonstrated capacity for sphere formation and clonal growth (essential features of stem cells). Remarkably the isolated ME-CSCs and cell isolated from auditory canal skin showed similar clonal efficiency. This is in contrast to Keratinocytes isolated from Cholesteatoma as described from Cheshire *et al*.^22^. In addition, ME-CSCs and ACSCS showed expression of neural crest stem cell (NCSC) markers Nestin and S100B and “stemness” markers c-myc and KLF-4, and were able to differentiate into both mesodermal and ectodermal lineage pathways, further demonstrating their stem cell characteristics. Cultivated ME-CSCs and ACSCs showed no changes in their genetic stability. These findings are also consistent with the normal DNA content measured in cells from cholesteatoma tissue by Desloge and colleagues^13^.

**Figure 6:**
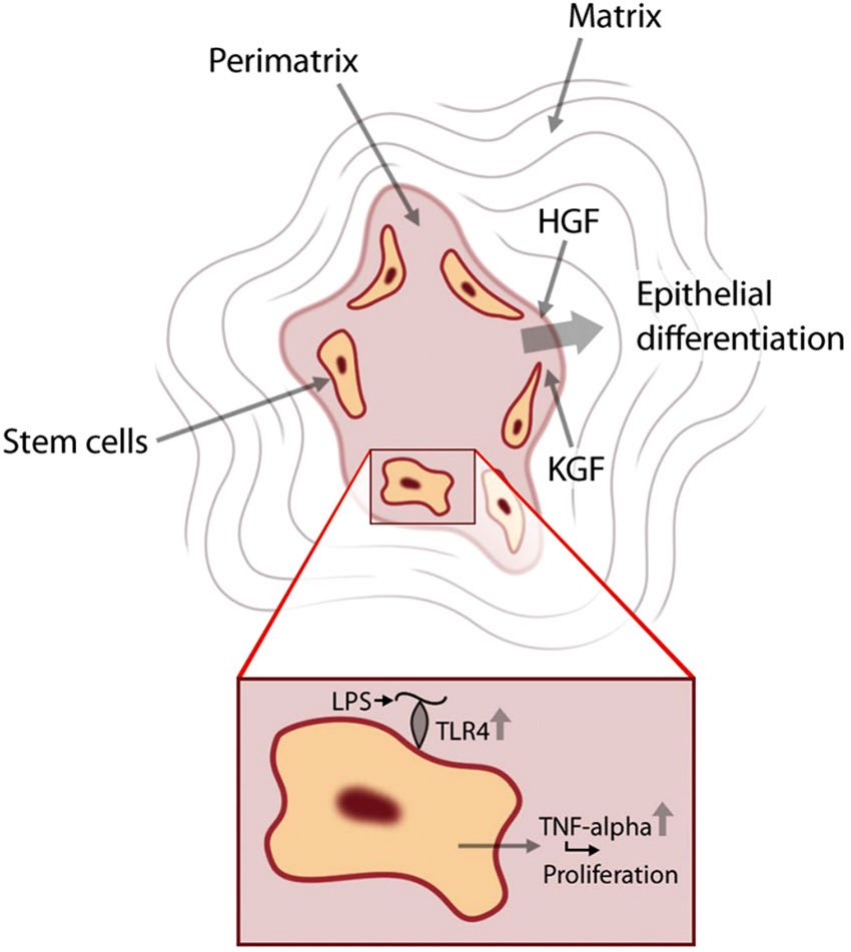
Schematic illustration of the contribution of the ME-CSCs to the progression of the acquired cholesteatoma. Schematic view on the acquired cholesteatoma comprised of a matrix, perimatrix and stem cells. Growth factors, like HGF and KGF already present in this pathological tissue have the potential to induce epithelial differentiation in the ME-CSCs residing in the cholesteatoma. Additionally, ME-CSCs will cause an increased inflammation upon stimulation with LPS derived from gram-negative bacteria in the infected cholesteatoma tissue.

Cholesteatoma tissue is highly inflamed and possesses inflammatory characteristics such as enhanced TLR4 expression^14^. Interestingly, these derived stem cells preserved their enhanced TLR4 expression and this putative increased sensitivity to inflammatory stimuli *in-vitro* may well contribute to the inflammatory environment *in-vivo* (inset Fig. 6).

Besides increased TLR4-expression, different cytokines are also known to be expressed in cholesteatoma tissue. EGF was described to be highly expressed in cholesteatoma compared to normal auditory canal skin^23^. Within cholesteatoma tissue, HGF is predominantly expressed in the perimatrix^24^ and is highly upregulated in cholesteatoma microenvironment compared to auditory canal skin^25^. The expression level of KGF was found to be increased in cholesteatoma tissue compared to healthy auditory canal skin, particularly in a strong inflammatory environment^26,27^. Interestingly, growth factors HGF^28^ and KGF^29^ were also shown to be able to promote epithelial differentiation. Proliferation of epidermal cells is particularly stimulated by EGF^30^, HGF^31^ and KGF^29^.

We have demonstrated that exposure of ME-CSCs to cytokines highly expressed in cholesteatoma tissue, leads to the expression of CK-14 and CK-18 at the mRNA and protein level. Hence these differentiated stem cells were named keratinocyte-like cells; and therefore, this suggests a potential contribution of ME-CSCs to the pathogenesis of middle ear cholesteatoma (Fig. 6). Indeed the increased expression of KGF and keratinocyte growth factor receptor (KGFR) could be correlated to the recurrence rate of cholesteatoma^32^. Furthermore, the transfection of KGF into a mouse model led to the formation of middle ear cholesteatoma^33^.

During development the middle ear is lined by neural crest derived cells (NCSCs). In mice especially, the attic is lined by NCSCs, which give rise to epithelial cells^34^. We have shown that cultivated ME-CSCs express neural crest markers (suggesting a neural crest-origin), and that these cells are potentially involved in cholesteatoma formation. Further, the presence and distribution of ME-CSCs within cholesteatoma tissue may be due to recruitment of ME-CSCs to the lesion. Interestingly Wang *et al*.^35^ and Kim *et al*.^36^ demonstrated the presence of epidermal stem cells in the tympanic membrane. Localization of these cells was accomplished by Knutsson *et al*. 2010, who located them in the umbo, the annular region and along the malleus^37^. All of these studies utilized the marker β1-integrin, an epidermal stem cell marker established by Jones *et al*. in 1993^38^. Furthermore, Rusu *et al*. could identify endothelial progenitor cells in the tympanic membrane using ultrastructural markers^39^. We verified the co-localization of β1-integrin with Nestin in the cholesteatoma tissue, whereas the auditory canal skin exhibited the regular β1-intergrin distribution (cf. Supplementary Figure S4), found in the epithelial layer reviewed in^40^. We therefore suggest that the stem cells described in this study might have their origin in the epithelial stem cell niche of the tympanic membrane and could contribute to the pathogenesis of cholesteatoma through differentiation into keratinocyte-like cells and by an enhancement of the inflammatory environment.

Our data add to the body of work from disease models established by other groups. We have shown that ME-CSCs are present within cholesteatoma, and are potentially involved in cholesteatoma formation, progression or both. We therefore propose a novel hypothesis in which stem cells might be involved in the complex pathogenesis of middle ear cholesteatomas. The discovery of this distinct stem cell population allows for a different perspective of cholesteatoma pathogenesis and may lead to more targeted therapies and new treatment strategies.

## Materials and Methods

### Ethics declaration

Acquired cholesteatomas and external auditory canal skin specimens were obtained from patients undergoing middle ear surgery at Klinikum Bielefeld Mitte (Bielefeld, Germany) after fully informed written consent according to local and international guidelines. The ethics board of the medical faculty of the University of Münster approved the procedures in this article (Az 2012–015-s-S). All experiments were performed in accordance with these approved guidelines and regulations.

### Source material and tissue preparation

Cholesteatoma tissue and external auditory canal skin were obtained from patients undergoing middle ear surgery at Klinikum Bielefeld Mitte (Bielefeld, Germany) after informed written consent according to local and international guidelines. After surgical removal, samples were used for cryostat sections or cut into small pieces, treated with Collagenase I (0.375 U/ml in PBS, SERVA Electrophoresis GmbH) for at least 1 hour at 37 °C and mechanically dissociated.

### Hematoxylin-eosin staining

Frozen 10 µm thick cryosections of cholesteatoma tissue and external auditory canal skin were subjected to H&E staining as described in^41^. Stained sections were microscopically examined using Olympus CKX41 (Olympus Deutschland GmbH, Hamburg, Germany).

### Alizarin red S staining

Stem cells were fixed for 20 min using 4% paraformaldehyde (PFA) and washed with PBS and subsequently with H_2_O. A staining solution of 1% Alizarin Red S (Waldeck) in H_2_O with a pH value of 4.3 was applied for 5 min at RT and imaged using CKX41 (Olympus Deutschland GmbH).

### Immunohistochemistry and Immunocytochemistry

Cryosections of cholesteatoma tissue and external auditory canal skin or cultivated cells were fixed for 20 min using 4% PFA followed by permeabilization in TritonX-100 (tissue: 0.2%, cells: 0.02%, Applichem) containing 5% goat serum for 30 minutes. Primary antibodies used were mouse anti-Nestin 1:200 (Millipore), rabbit anti-S100B 1:100 (Dako) for stem cell detection already utilized in Hauser *et al*.^15^. Additional primary antibodies were mouse anti-β-III-tubulin 1:100 (Promega), rabbit anti-MAP2 1:100 (Santa Cruz Biotechnology), mouse anti-TLR4 1:100 (Acris Antibodies GmbH), rabbit ant-β1-integrin (Sigma Aldrich), mouse anti-CK-14 1:200 (Millipore) and mouse anti-CK-18 1:800 (Cell Signaling Technology). They were applied for 1 h (cells) or 2 h (sections) at RT. Secondary fluorochrome-conjugated antibody 1:300 (Alexa 555 anti-mouse or Alexa 488 anti-rabbit, Invitrogen, Life Technologies GmbH) were subsequently applied for 1 h at RT. Nuclear counterstaining was performed using 4,6-Diamidin-2-phenylindol (DAPI, 1 µg/ml) for 15 minutes at RT followed by mounting with Mowiol. Imaging and analysis was performed using confocal laser scanning microscope (CLSM 780, Carl Zeiss) with ZEN software (Carl Zeiss).

### Cultivation of cholesteatoma and external auditory canal skin derived cells

Cholesteatoma- and external auditory canal skin-tissue dissociated as described above was centrifuged at 300 × g for 10 minutes and pre-cultivated in surface treated T-25 cell culture flasks (Sarstedt AG & Co.) in a humidified incubator (Binder) at 37 °C and 5% CO_2_ in standard medium (DMEM/F-12 (Sigma-Aldrich) containing Penicillin, Streptomycin, Amphotericin B (Sigma-Aldrich), EGF (20 ng/ml; Peprotech), bFGF (also known as FGF2 or FGF-β 40 ng/ml, Peprotech) and B27 supplement (Gibco, Life Technologies) with addition of 10% human blood plasma. Subsequently cultivated cells were transferred to T-25 low adhesion cell culture flasks (Sarstedt) using standard medium with additional heparin (2 µg/ml, Sigma-Aldrich) for cultivation of free-floating spheres at 37 °C and 5% CO_2._ This step ensured the purification of the middle ear cholesteatoma-derived stem cells (ME-CSCs) and auditory canal skin stem cells (ACSCs). Purified ME-CSCs and ACSCs were re-cultivated in surface treated T-25 cell culture flasks (Sarstedt AG & Co.) using the standard medium with 10% human blood plasma as described for nasal stem cells^15,16^. Cells were fed every two days. For passaging, cells were treated with Collagenase I (0.375 U/ml in PBS, SERVA) for at least 30 minutes at 37 °C followed by centrifugation at 300 × g for 10 minutes and cultivation as described above. Freezing and thawing of ME-CSCs and ACSCs was performed as described for nasal stem cells^15,16^.

### Cultivation of induced pluripotent stem cells

Multipotent adult human stem cells^15^ were transduced with a polycystronic lentiviral vector comprising Oct4, Sox2, Klf4 and c-Myc. Pluripotency was previously validated by robust expression of Oct4, Sox2, Lin28 and Nanog as well as differentiation into cell types of all three germ layers *in vitro* and *in vivo*. Induced pluripotent stem cells were expanded by cultivation in E8 medium (E8) without supplements (Stem Cell Technologies) in vitronectin coated 6 wells plates without feeder cells. The medium was changed every day.

### Reverse transcriptase PCR

RNA was isolated from ME-CSCs and ACSCs using innuPREP RNA Mini Kit (Analytic Jena AG) according to manufacturer’s guidelines. RNA quality and concentration was assessed using a Nanophotometer (Implen GmbH). For cDNA synthesis M-MuLV RT DNA-Polymerase (Bio-Budget Technologies GmbH) was applied according to the manufacturer’s guidelines. PCR was performed with the 5 × Hot-Start Taq PCR-Mastermix (Bio-Budget Technologies GmbH) according to the manufacturer’s guidelines.

### Real-time qPCR

Total RNA isolation and cDNA synthesis were performed as described above. For qPCR, technical triplicates were prepared using 5× EvaGreen QPCR-Mix (Bio-Budget Technologies GmbH) according to manufacturer’s guidelines and analyzed via a Magnetic Induction Cycler (bio molecular systems) using micPCR software (bio molecular systems). GraphPad Prism software (GraphPad Software) was used for statistical analyses. Primer sequences are depicted in Table 1.

**Table 1:** Primer sequences.

Target	Forward primer sequence	Reverse primer sequence
GAPDH	ATCGTGGAAGGACTCATGACCACA	TTTTCTAGACGGCAGGTCAGGT
S100B	GGAGGTTGTGGACAAAGTCATGGA	TCAAAGAACTCGTGGCAGGCAGTA
TLR4	CACAGACTTGCGGGTTCTACATCA	TGGACTTCTAAACCAGCCAGACCT
LCN2	TGTCACCTCCGTCCTGTTTAGGAA	ACTCTTAATGTTGCCCAGCGTGAA
TNF-α	AAGCCCTGGTATGAGCCCATCTAT	AGGGCAATGATCCCAAAGTAGACC
A20	TACCCTTGGTGACCCTGAAG	CCTTGGACGGGGATTTCTAT
CK18	GACCGCCTGGCCTCTTAC	ACCTGGGGTCCCTTCTTCTC
CK14	CCTCCTCCAGCCGCCAAATCC	TTGGTGCGAAGGACCTGCTCG
Klf4	TCTCCAATTCGCTGACCCATCCT	TTCAGCACGAACTTGCCCATCA
Oct4	GCTTGGAGACCTCTCAGCCT	TGATGTCCTGGGACTCCTC
c-Myc	AAGACTCCAGCGCCTTCTCT	CTCTGACCTTTTGCCAGGAG
Sox2	TGCAGTACAACTCCATGACCA	GTGCTGGGACATGTGAAGTCT

### LPS-Stimulation of cultivated ME-CSCs and ACSCs

To simulate inflammation *in-vitro*, ME-CSCs and ACSCs were treated with 100 ng/ml LPS (rough strains from *Salmonella enterica* Re 595, cat. No. L9764, Sigma-Aldrich) dissolved in DMEM high glucose with additional L-Glu, Penicillin, Streptomycin and 10% FCS. The cells were stimulated for 6 h, followed by RNA isolation, cDNA synthesis and real-time qPCR analysis. ME-CSCs and ACSCs treated only with the medium described above served as control.

### Flow cytometric measurement of DNA content

For analyzing the DNA content of ME-CSCs the CyStain PI absolute T Kit (Partec) was applied according to manufacturer’s guidelines. Samples were analyzed by the CyFlow space flow cytometer (Partec) using FloJo software (Tree Star).

### Clonal density assay

To investigate if ME-CSCs can grow at single cell level, cells were cultivated using standard medium comprising 10% blood plasma in a limited dilution assay as described in^15^. Dissociated cells were diluted to 1 cell/100 µl in standard medium containing 10% human blood plasma medium and placed into U-bottom 96-well plates. Two hours after plating, wells were analyzed for the presence of single cells. Clonal growth of cells was assessed using an Olympus CKX41 microscope (Olympus Deutschland GmbH).

### Differentiation into osteoblasts

To induce osteogenic differentiation, the ME-CSCs and ACSCs seeded in DMEM containing 10% FCS (Sigma-Aldrich) at a density of 3 × 10^3^ cells/cm^2^. After 48 h the medium was changed to an osteogenic induction medium containing 100 nM dexamethasone (Sigma-Aldrich), 10 mM β-glycerophosphate (Sigma-Aldrich) and 0.05 mM L-ascorbic acid-2-phosphate (Sigma-Aldrich). Medium was changed every two to three days, after 21 days Alizarin red S staining was performed as described above.

### Induced neuronal differentiation

For induced neuronal differentiation cultivated ME-CSCs and ACSCs were re-suspended in DMEM (Sigma-Aldrich) containing 10% FCS (Sigma-Aldrich) and plated at a density of 5 × 10^4^ cells per 12-well. After 48 h, neural differentiation was induced as described in^42^. After 28 days, immunocytochemical stainings were performed as described above.

### Differentiation into epithelial lineage

To study whether ME-CSCs can differentiate into keratinocyte-like cells, they were plated at a density of 3 × 10^3^ cells/cm² in a 12-well and cultivated in DMEM low glucose (Sigma-Aldrich) containing 10% FCS, keratinocyte growth factor (KGF, 10 ng/ml; Peprotech) and epidermal growth factor (EGF, 20 ng/ml; Peprotech). After 3 days of culture, hepatocytes growth factor (HGF, 10 ng/ml; Peprotech) and insulin-like growth factor-2 (IGF-II, 60 ng/ml; Peprotech) were added. ACSCs cells served as control. After 14 days, RNA isolation and immunocytochemical stainings were performed as described above.

## Conclusion

We demonstrate here for the first time the presence of stem cell populations in cholesteatoma tissue and auditory canal skin. Our findings indicate new aspects of the complex biology of cholesteatoma, suggesting ME-CSCs may be involved in the pathogenesis of the cholesteatoma and may play an important role in its progression. ME-CSCs may further serve as a promising *in vitro* model in terms of pharmacological research, facilitating innovative treatment strategies.

## Electronic supplementary material

Dataset1
