# Supplementary material for: Stem cells in middle ear cholesteatoma contribute to its pathogenesis
Source: Sci Rep. 2018 Apr 18;8:6204. doi: 10.1038/s41598-018-24616-4 (PMC5906547; doi:10.1038/s41598-018-24616-4)
Supplement: Supplementary file 1 — Dataset1 [file 41598_2018_24616_MOESM1_ESM.docx]

**Stem cells in middle ear cholesteatoma contribute to its pathogenesis**

Julia Nagel^1,2,^* , Saskia Wöllner^1,2,^*, Matthias Schürmann^1^, Viktoria Brotzmann^1^, Janine Müller^1^, Johannes FW Greiner^1,2^, Peter Goon^3^, Barbara Kaltschmidt^2,4^, Christian Kaltschmidt^2^ and Holger Sudhoff^1,#^

^1^ Department of Otolaryngology, Head and Neck Surgery, Klinikum Bielefeld, 33604 Bielefeld, Germany

^2^ Department of Cell Biology, University of Bielefeld, 33619 Bielefeld, Germany

^3^ Department of Dermatology, Norfolk and Norwich University Hospital, Colney Lane, Norwich, NR4 7UY, UK

^4^ AG Molecular Neurobiology, University of Bielefeld, 33619 Bielefeld, Germany

*Authors have equal contributions

^#^Corresponding Author:

Professor Holger Sudhoff, PhD, MD, FRCS, FRCPath

Department of Otolaryngology, Head and Neck Surgery

Klinikum Bielefeld, Teutoburger Str. 50

33604 Bielefeld, Germany

Tel. + 49 521 581 3301

FAX + 49 521 581 3399

[holger.sudhoff@rub.de](mailto:holger.sudhoff@rub.de)

# Supplementary Information


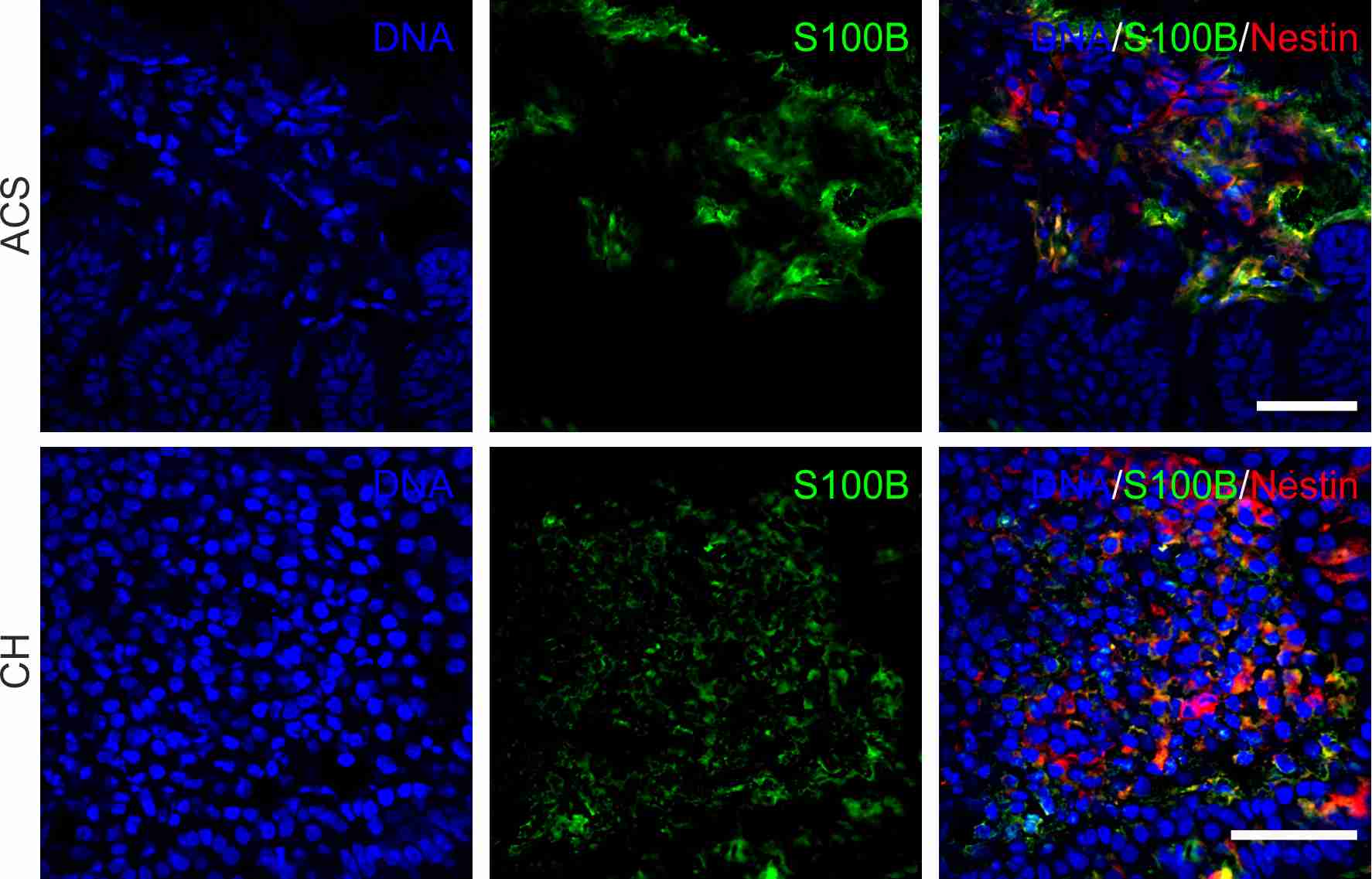


**Supplementary figure S1.** The co-localization of S100B and Nestin in cholesteatoma and auditory canal skin. Immunohistochemical staining of cryo-sectiones revealed the presence of cells co-expressing S100B and Nestin in cholesteatoma as well as in auditory canal skin tissue. Scale bar: 50µm.


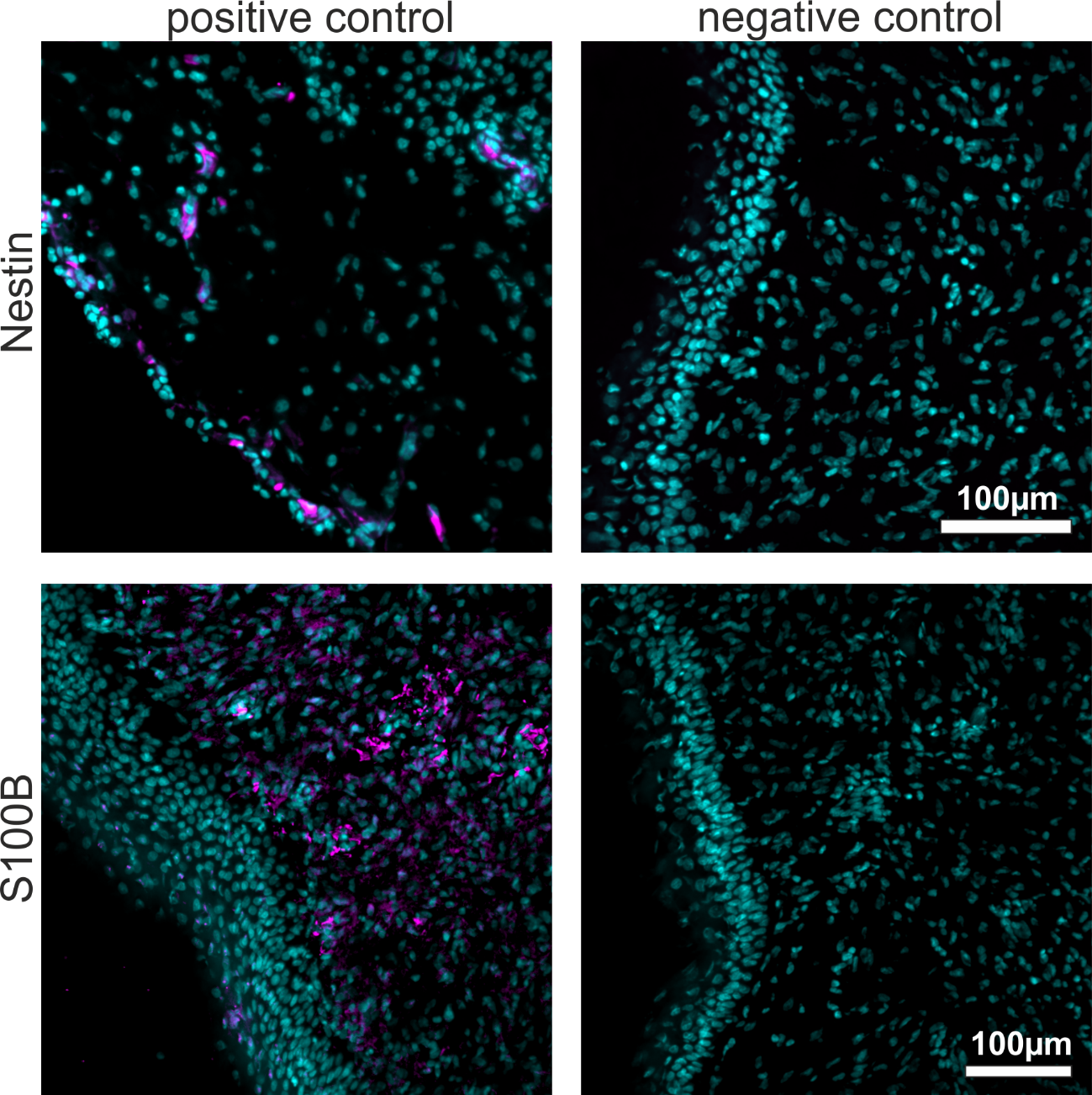


**Supplementary figure S2.** Positive and negative control of Nestin and S100B immunohistochmistry in cholesteatoma tissue. Scale bar: 50µm.


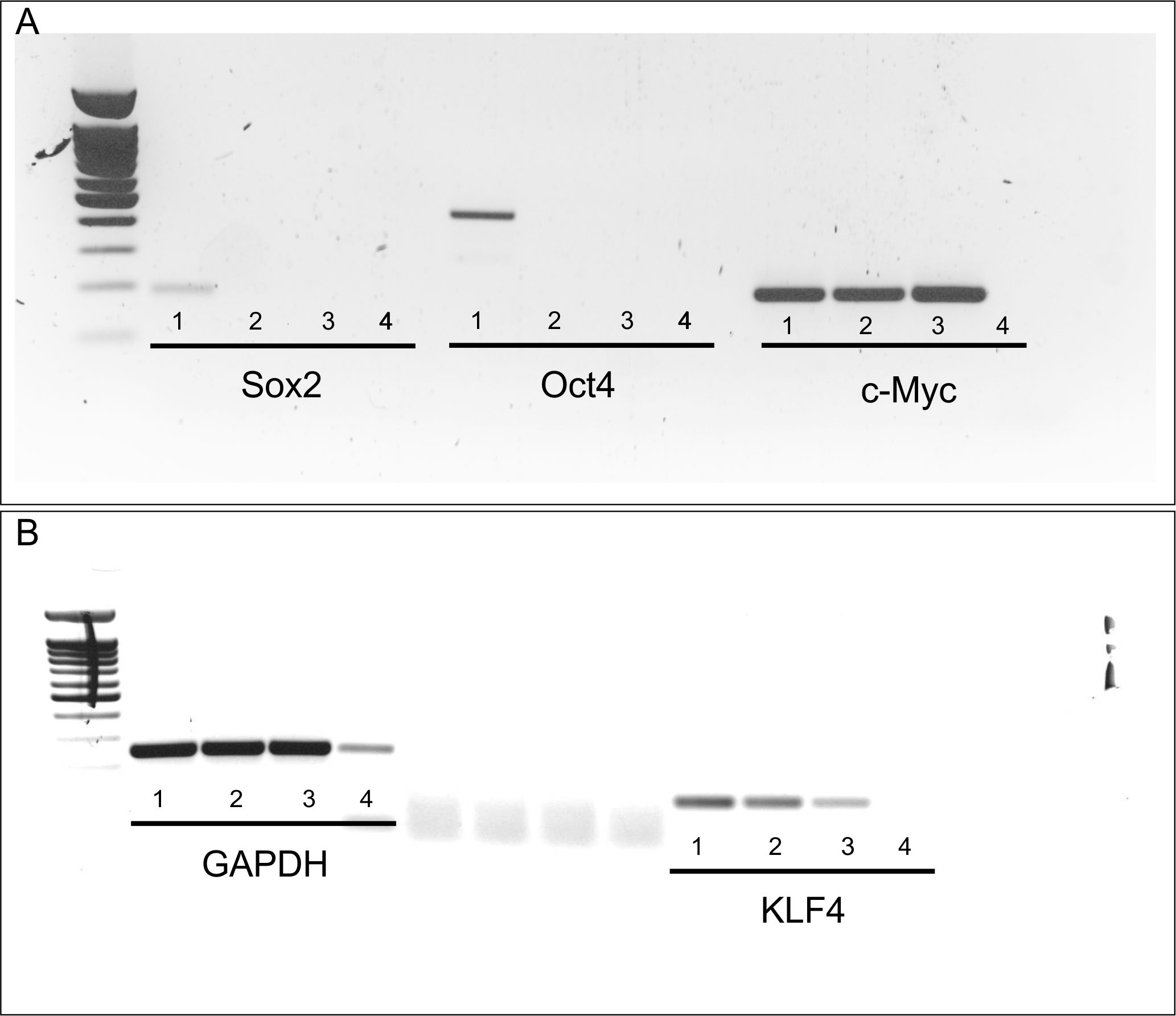


**Supplementary figure S3.** Gel blot of RT-PCR of the pluripotent factors. **A** RT-PCR of Sox2, Oct4 and c-Myc. The simple numbering is 1: PT: Positive control, iPSCs, 2: ME-CSCs, 3: ACSCs, 4: NT, No template control. **B** RT-PCR of GAPDH and KLF4.


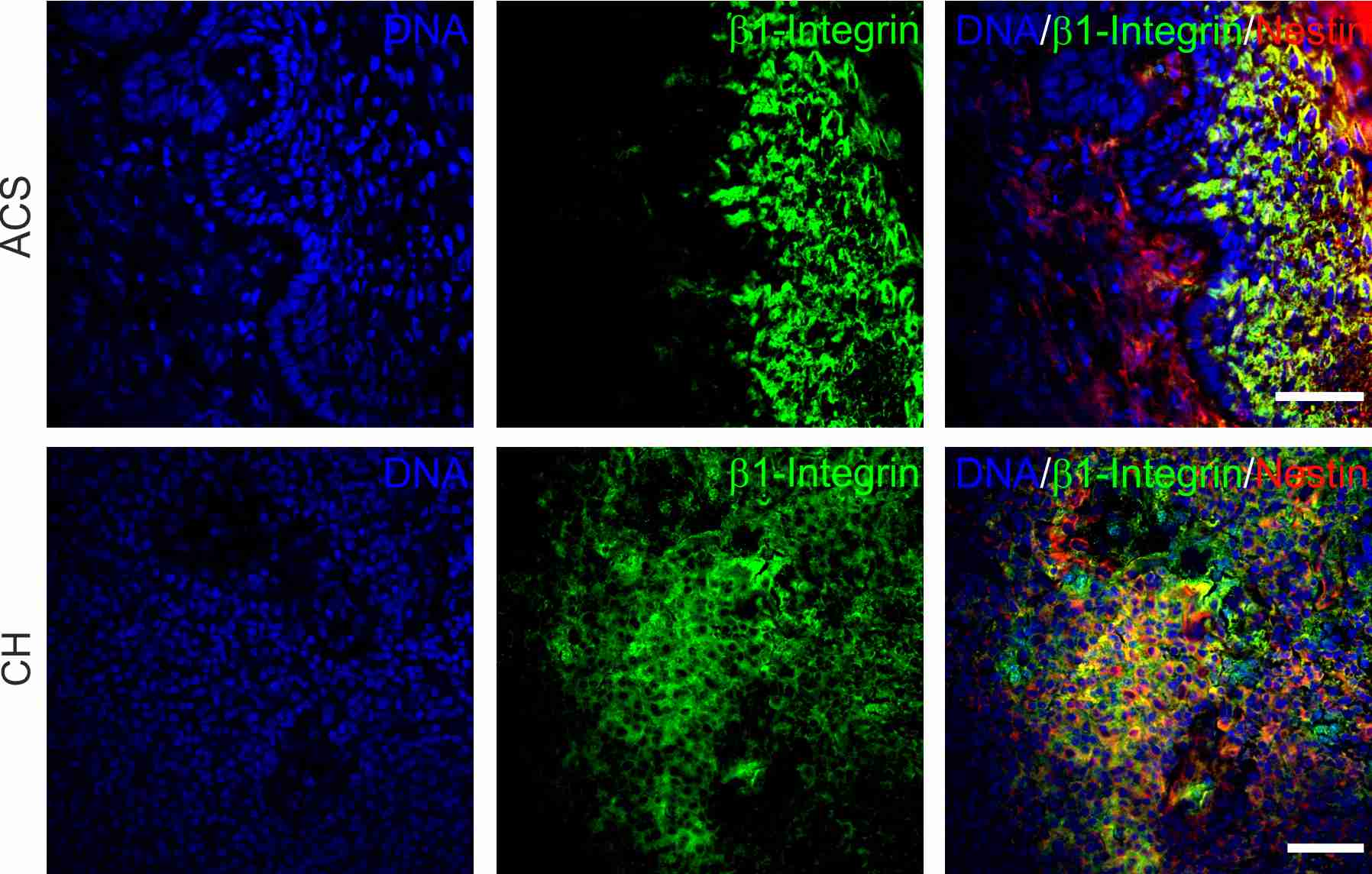


**Supplementary figure S4.** Co-expression of β1-integrin and Nestin in the cholesteatoma tissue. Immunohistological staining of cholesteatoma tissue (CH) and auditory canal skin (ACS). In the auditory canal skin the β1-integrin expressing cells are separated by the basal layer from the Nestin expressing cells in the lamina propria. In the cholesteatoma tissue β1-integrin shows a high degree of co-localization with Nestin. Scale bar: 50µm.
